# Roles of taurine-mediated tonic GABA_A_ receptor activation in the radial migration of neurons in the fetal mouse cerebral cortex

**DOI:** 10.3389/fncel.2014.00088

**Published:** 2014-03-28

**Authors:** Tomonori Furukawa, Junko Yamada, Tenpei Akita, Yoshitaka Matsushima, Yuchio Yanagawa, Atsuo Fukuda

**Affiliations:** ^1^Department of Neurophysiology, Hamamatsu University School of MedicineHamamatsu, Shizuoka, Japan; ^2^Department of Neurophysiology, Hirosaki University Graduate School of MedicineHirosaki, Aomori, Japan; ^3^Department of Chemistry, Hamamatsu University School of MedicineHamamatsu, Shizuoka, Japan; ^4^Department of Genetic and Behavioral Neuroscience, Gunma University Graduate School of MedicineMaebashi, Gunma, Japan

**Keywords:** taurine, GABA_A_ receptor, tonic current, radial migration, non-vesicular release, volume-sensitive anion channel, GAD67, taurine transporter

## Abstract

γ-Aminobutyric acid (GABA) depolarizes embryonic cerebrocortical neurons and continuous activation of the GABA_A_ receptor (GABA_A_R) contributes to their tonic depolarization. Although multiple reports have demonstrated a role of GABA_A_R activation in neocortical development, including in migration, most of these studies have used pharmacological blockers. Herein, we performed *in utero* electroporation in GABA synthesis-lacking homozygous GAD67-GFP knock-in mice (GAD67^GFP/GFP^) to label neurons born in the ventricular zone. Three days after electroporation, there were no differences in the distribution of labeled cells between the genotypes. The dose–response properties of labeled cells to GABA were equivalent among genotypes. However, continuous blockade of GABA_A_R with the GABA_A_R antagonist SR95531 accelerated radial migration. This effect of GABA_A_R blockade in GAD67^GFP/GFP^ mice suggested a role for alternative endogenous GABA_A_R agonists. Thus, we tested the role of taurine, which is derived from maternal blood but is abundant in the fetal brain. The taurine-evoked currents in labeled cells were mediated by GABA_A_R. Taurine uptake was blocked by a taurine transporter inhibitor, 2-(guanidino)ethanesulfonic acid (GES), and taurine release was blocked by a volume-sensitive anion channel blocker, 4-(2-butyl-6,7-dichlor-2-cyclopentylindan-1-on-5-yl) oxobutyric acid, as examined through high-performance liquid chromatography. GES increased the extracellular taurine concentration and induced an inward shift of the holding current, which was reversed by SR95531. In a taurine-deficient mouse model, the GABA_A_R-mediated tonic currents were greatly reduced, and radial migration was accelerated. As the tonic currents were equivalent among the genotypes of GAD67-GFP knock-in mice, taurine, rather than GABA, might play a major role as an endogenous agonist of embryonic tonic GABA_A_R conductance, regulating the radial migration of neurons in the developing neocortex.

## INTRODUCTION

During the development of the nervous system, cortical neurons are generated in the ventricular zone (VZ; Sidman and Rakic, 1973; Caviness and Rakic, 1978). After terminal mitosis, neurons derived from the VZ migrate radially through the cell-sparse intermediate zone (IZ) into the cortical plate (CP) to give rise to glutamatergic neurons of the neocortex, such as pyramidal and stellate neurons (Tan et al., 1998; Andjelic et al., 2009). During the early developmental stage of cortical neurons, functional GABA_A_ receptors (GABA_A_R) are present on both proliferative and early postmitotic cells (LoTurco et al., 1995; Owens et al., 1996). Furthermore, GABA_A_R activation can regulate the migration of neuronal precursors (Komuro and Rakic, 1998; Nadarajah and Parnavelas, 2002; Marin and Rubenstein, 2003). In the developing cerebral cortex, activation of GABA_A_R modulates radial migration as a stop signal (Behar et al., 1998; Heck et al., 2007). GABA_A_Rs on neural precursor cells in the postnatal subventricular zone (SVZ) are activated tonically, which reduces the migration speed of the cells (Bolteus and Bordey, 2004; Bolteus et al., 2005). Tonic activation of GABA_A_Rs is also evident in embryonic neurons before synapse formation has occurred (Valeyev et al., 1993; LoTurco et al., 1995; Owens et al., 1999; Demarque et al., 2002). The effects of GABA_A_R activation on neuronal migration have been described using GABA_A_R antagonists in cultured brain slices and dissociated cells (Behar et al., 1996, 1998, 2000; Bolteus and Bordey, 2004). However, most reports have only focused on the role of GABA_A_R in cortical cell migration assuming that under natural conditions, these receptors are activated by GABA. However, this hypothesis has never been directly tested, and the roles of other endogenous GABA_A_R agonists have not been addressed.

GABA is one of the earliest neurotransmitters detectable in the developing central nervous system (CNS; Lauder et al., 1986; Behar et al., 1993; Ma and Barker, 1995) and is synthesized from glutamic acid by glutamic acid decarboxylase (GAD). The GAD67 isoform of GAD is expressed in GABAergic interneurons. GAD67-expressing cells originating from ganglionic eminences migrate tangentially and become distributed in the developing cerebral cortex (Tamamaki et al., 2003). It is generally thought that GABA from GAD67-expressing cells activates GABA_A_Rs and plays an important role in radial migration. However, the gross structure of the brain appears normal in new-born GAD67 knock-out mice (Asada et al., 1997; Ji et al., 1999), which is inconsistent with a role of GABA in radial migration. Thus, it is also possible that the GABA_A_Rs of radially migrating cells may be activated by endogenous ligands other than GABA.

In the CNS, taurine (2-aminoethanesulfonic acid) is the most abundant amino acid (Kontro et al., 1984; Huxtable, 1989; Benitez-Diaz et al., 2003). Taurine is an endogenous partial agonist of the GABA_A_R and induces chloride currents in neurons (Linne et al., 1996; Ye et al., 1997). Although the capacity to synthesize taurine is low during fetal life (Hayes and Sturman, 1981; Kuo and Stipanuk, 1984; Ghisolfi, 1987), taurine levels are higher in the immature brain than in the adult brain (Kaczmarek, 1976; Benitez-Diaz et al., 2003). In mammals, sufficient taurine for embryonic development is received from the mother via the placenta, while neonates obtain taurine from their mothers’ milk (Sturman et al., 1977; Sturman, 1981). Taurine exhibits physiological actions as a trophic factor and as a neuromodulator during the development of the CNS (Sturman, 1993; Oja and Saransaari, 1996; Hernandez-Benitez et al., 2012). In taurine-deficient kittens, the migration of granule cells from the external granule cell layer to the inner layers of the cerebellum is delayed (Sturman et al., 1985). Although the physiological properties of taurine in the developing brain have been described, it is not yet known whether endogenous taurine activates GABA_A_Rs and modulates radial migration in the developing cerebral cortex.

In the present study, we hypothesized that taurine activates GABA_A_R tonically and affects radial migration of neurons in the developing cerebral cortex. Similar to GAD67 knock-out mice, GABA levels are decreased in heterozygous (GAD67^+^^/GFP^) and homozygous (GAD67^GFP/GFP^) GAD67-GFP knock-in mice (Tamamaki et al., 2003; Morishima et al., 2010). Furthermore, taurine synthesis can be suppressed via the intraperitoneal injection of D-cysteinesulfinic acid (D-CSA; Weinstein et al., 1988), and it has been reported that a low maternal taurine level results in low taurine levels in the fetus (Aerts and Van Assche, 2002). Thus, using GAD67-GFP knock-in mice and D-CSA, we examined the effects of the taurine activation of GABA_A_R on radial migration in the developing mouse cerebral cortex. Our results provide evidence of taurine-mediated tonic activation of GABA_A_Rs and reveal that taurine retards radial migration in the developing mouse cerebral cortex.

## MATERIALS AND METHODS

### ETHICAL APPROVAL

All experiments conformed to the guidelines issued by the Hamamatsu University School of Medicine for the ethical use of animals for experimentation, and all efforts were made to minimize the number of animals used and their suffering.

### ANIMALS

The generation of GAD67-GFP (Δneo) mice has been described previously (Tamamaki et al., 2003). In the present study, female heterozygous (GAD67^+^^/GFP^) mice were placed overnight with male (>9 weeks) GAD67^+^^/GFP^ mice in a cage under a 12-h light–dark cycle (lights off from 19:00 to 07:00). The day on which a virginal plug was observed in the morning was designated as embryonic day 0.5 (E0.5). Polymerase chain reaction (PCR) amplification was performed to discriminate the genotypes of individuals. For some experiments, we used also Wistar rats purchased from Japan SLC (Hamamatsu, Japan).

### *IN UTERO* ELECTROPORATION

Cells were transfected *in vivo* through *in utero* electroporation, as described previously (Inoue et al., 2012). Briefly, plasmids carrying monomeric red fluorescent protein (mRFP) downstream of a CAG promoter (Addgene, MA, USA) were prepared using the EndoFree Plasmid Kit (Qiagen, Hilden, Germany). Pregnant mice and rats were anesthetized with sodium pentobarbital (50 mg/kg intraperitoneally) at E14.5 and E15.5, respectively, and their uterine horns were exposed. Plasmid DNA was dissolved in phosphate buffered saline (PBS) at a final concentration of 0.5 μg/μl with Fast Green (final concentration 0.05% [v/v]). Plasmids were injected into the lateral ventricle using a glass micropipette and a controlled pipette system (IM-30, Narishige, Tokyo, Japan). The micropipettes were generated from glass capillaries (outer diameter 1.0 mm; Harvard Apparatus, South Natick, MA, USA) that were pulled using a P-97 micropipette puller (Sutter Instrument Co., Novato, CA, USA). Electric pulses were produced by an electroporator (CUY21EDIT; NepaGene, Ichikawa, Japan) and delivered by a round plate forceps-type electrode with a 5-mm diameter (CUY650P5; NepaGene). Electric pulses (43 V, 50 ms) were applied five times at intervals of 950 ms. The uterine horns were then returned to the abdomen.

### IMPLANTATION OF PLGA FOR SUSTAINED DRUG ADMINISTRATION *IN VIVO*

Copoly(d,l-lactic/glycolic acid) (PLGA) exhibits high bio-affinity and biodegradability and is therefore a useful material for achieving sustained drug release (Miller et al., 1977). PLGA (20 mg; Wako, Osaka, Japan) was dissolved in 100 μl of *N*-methyl-2-pyrrolidone (Wako; Kranz et al., 2001), and 10 μl of 10 mM SR95531 or water (Control) was added. Following *in utero* electroporation, the PLGA solution or SR95531-adsorbed PLGA (0.5 μl) was injected into the lateral ventricles of fetuses.

### ANALYSIS OF RADIAL MIGRATION BASED ON DISTRIBUTION PATTERNS OF NEURONS

Fetuses were killed at E17.5 and decapitated, and their brains were dissected out. The brains were fixed in 4% paraformaldehyde for 3 h at 4°C and then transferred to 30% sucrose phosphate buffer (0.1 M, pH 7.4) and left immersed for 3 days. The brains were then sectioned coronally at a thickness of 30 μm using a cryostat and counterstained with 4′,6-diamidino-2-phenylindole (DAPI) to indicate proliferative zones, after which the sections were transferred to slides and coverslipped. Images were subsequently captured using a cooled charge-coupled device (CCD) camera (Orca ER-G; Hamamatsu photonics, Hamamatsu, Japan) attached to an epifluorescence microscope (BX-51; Olympus, Tokyo, Japan). The E17.5 neocortex is laminated into the marginal zone (MZ), CP, subplate (SP), IZ, and SVZ/VZ (Shinozaki et al., 2007). Based on the cytoarchitecture revealed by DAPI counterstaining, the regions with abundant cells were considered the SVZ/VZ and CP. The IZ and SP were defined as the regions between these areas (Caric et al., 1997; Inoue et al., 2012). The boundary between the IZ and SP was assessed based on the DAPI signal density, which was higher in the IZ than the SP. To determine the distribution pattern of migrating neurons, all of the red fluorescent protein (RFP)-positive cells in the cortex of each section were counted. The area in which GFP-positive cells were counted was approximately 300 μm wide and included the full thickness of the cortex. The numbers of RFP- or GFP-positive cells in the MZ, CP, SP, IZ, and SVZ/VZ were counted using ImageJ software (National Institutes of Health, Bethesda, MD, USA). The RFP- or GFP-positive cells in each area were counted and are presented as the ratio to the number of counted cells in a slice.

### HISTOLOGY AND IMMUNOHISTOCHEMISTRY

Taurine localization was observed immunohistochemically. The fetal neocortex of E17.5 mice was fixed via perfusion with 4% paraformaldehyde/0.5% glutaraldehyde in 0.1 M Tris buffer (TB). The brains were removed and immersed in the same fixative for 3 h at 4°C. After post-fixing, the brains were transferred to 15% sucrose TB for 1 h and 30% sucrose TB for 3 days. Tissues were cut coronally with a cryostat into sections with a thickness of 30 μm and rinsed in Tris-buffered saline (TBS). The sections were first treated with 0.5% NaBH_4_, then incubated for 0.5 h in 0.3% H_2_O_2_ in TBS and subsequently with an antibody against taurine (TT100, polyclonal rabbit IgG; Signature Immunologics, Salt Lake City, UT, USA) for 36 h at 4°C. After rinsing in TBS, the sections were incubated for 1 h with a secondary antibody against rabbit IgG (anti-rabbit biotinylated, BA1000; Vector Laboratories, Burlingame, CA, USA). The primary and secondary antibodies were diluted in TBS containing 2% horse serum albumin and 0.1% Triton X-100. Visualization was performed using the Biotin/Avidin system (Vector Laboratories). Sections were treated in diaminobenzidine (DAB). After rinsing, the sections were mounted on slides and coverslipped and finally observed under a microscope (AX-80; Olympus) equipped with a CCD camera (EP-70; Olympus).

### ELECTRON MICROSCOPY

After deeply anesthetizing the animals via hypothermia and perfusing them transcardially with 4% paraformaldehyde/0.5% glutaraldehyde in 0.1 M 4-(2-hydroxyethyl)-1-piperazineethanesulfonic acid (HEPES) buffer, their brains were removed and post-fixed for 10 min. Coronal sections (50 μm) were cut with a vibratome (DTK-1500; Dosaka, Kyoto, Japan) in the same fixative solution. The sections were incubated in 0.1 M HEPES buffer for 1 day at 4°C. After rinsing in PBST (0.1% Triton X-100/0.1 M PBS) three times, the sections were incubated for 1 h in blocking buffer containing 2% horse serum albumin and 0.1% Triton X-100, then incubated for 36 h at 4°C with a polyclonal antibody against taurine (Signature Immunologics). The sections were subsequently washed with PBS three times, incubated in blocking buffer for 1 h at room temperature, and then treated with an anti-rabbit secondary antibody coupled with 1.4-nm diameter gold particles (Nanogold; Nanoprobes, Yaphank, NY, USA) overnight at room temperature. After this step, an immunoelectron microscopic analysis was performed by Tokai Electron Microscopy Inc. (Nagoya, Japan). After the sections were washed with PBS and fixed in 2% glutaraldehyde in 0.1 M sodium cacodylate buffer (pH 7.4) for 3 h at room temperature, the gold particles were enlarged for microscopic examination with the HQ-Silver Enhancement Kit (Nanoprobes) and routinely processed for electron microscopic examination. The embedded samples were sectioned (thickness 80 nm) with an ultra-microtome (LKB2088; LKB, Bromma, Sweden) and stained with uranyl acetate and lead citrate. The sections were then carbon-coated in a vacuum and observed with a JEOL transmission electron microscope (JEM 2000EX; JEOL, Tokyo, Japan) at 100 kV.

### ACUTE SLICE PREPARATION

Coronal cortical slices were obtained from mouse fetuses at E17.5 (or rat fetuses at E18.5). The maternal mice or rats were deeply anesthetized via inhalation of halothane, and the fetal brains were rapidly removed and placed in cold (4°C), oxygenated, modified artificial cerebrospinal fluid (ACSF). The solution contained the following components (in mM): 220 sucrose, 2.5 KCl, 1.25 NaH_2_PO_4_, 10.0 MgSO_4_, 0.5 CaCl_2_.2H_2_O, 26.0 NaHCO_3_, and 30.0 glucose (330–340 mOsm). Coronal slices (400 μm) were cut in the modified ACSF using a vibratome (VT-1000; Leica, Wetzlar, Germany). The slices were maintained in oxygenated standard ACSF consisting of (in mM) 126 NaCl, 2.5 KCl, 1.25 NaH_2_PO_4_, 2.0 MgSO_4_, 2.0 CaCl_2_, 26.0 NaHCO_3_, and 20.0 glucose gassed with 95% O_2_–5% CO_2_ at room temperature prior to recording. Individual slices were transferred to a recording chamber, perfused (2 ml/min) with pre-gassed standard ACSF, and maintained at 30°C.

### ELECTROPHYSIOLOGY

In all of the electrophysiological experiments, D-(-)-2-amino-5-phosphonopentanoic acid (D-AP5, 50 μM), 6-cyano-7-nitroquinoxaline-2,3-dione (CNQX, 10 μM), CGP55845 (3 μM), and tetrodotoxin (TTX, 0.5 μM) were added to the ACSF. Patch electrodes were fabricated from 1.5-mm diameter borosilicate capillary tubing (GD-1.5; Narishige) using a P-97 horizontal puller (Sutter Instruments). The electrode resistance ranged from 7 to 10 MΩ when the electrode was filled with a solution containing (in mM) 110 CsCl, 6 MgCl_2_, 10 ethylene glycol tetraacetic acid (EGTA), 10 HEPES, 4 K_2_-ATP, 20 K_2_-phosphocreatine, and 50 U creatine phosphokinase. The pH was adjusted to 7.3 with CsOH. Under these experimental conditions, GABAergic IPSCs (inhibitory postsynaptic currents) reversed at approximately 0 mV (the E_Cl_ predicted by the Nernst equation). With the cells voltage-clamped at -60 mV, the GABAergic IPSC appeared as an inward current. The recorded currents were filtered at 2 kHz and digitized at 1–10 kHz using DigiData1322A and pCLAMP9 software (Molecular Devices, Sunnyvale, CA, USA). GABA, taurine, and glycine were added through another pipette to the recorded cell soma using a puffer application system (IM-300; Narishige). The data were analyzed using Clampfit9 (Molecular Devices). Dose–response relationships for GABA were fitted to the Hill equation: $I=I_{\max}/\left\{1+{\left[E\,C_{50}/\left(G\,A\,B\,A\right)\right]}^{n}\right\},$ where *I* is the peak current measured at a given concentration of GABA; *I*_max_ is the current at the maximal GABA concentration; the EC_50_ is the half-maximal effective GABA concentration; and *n* is the Hill coefficient. Ligand applications were separated by 3 min intervals to allow recovery from desensitization when present.

### MEASUREMENT OF AMBIENT GABA AND TAURINE

Ambient GABA and taurine levels were measured using high-performance liquid chromatography (HPLC) methods. Following the preparation of tissue slices, the cerebral cortex was cut from two coronal slices. The cortices were equilibrated for 1 h in a holding chamber filled with continuously oxygenated ACSF. In taurine loading experiments, 1 or 10 mM taurine was dissolved in the ACSF. After 1 h of incubation with or without taurine, the cortices were rinsed thoroughly and placed in a submerged-type small chamber filled with ACSF that was oxygenated and filtered (Minisart; Sartorius, Göttingen, Germany) and incubated for 1 h at 30°C. Hypotonic medium consisting of (in mM) 31 NaCl, 2.5 KCl, 1.25 NaH_2_PO_4_, 2.0 MgSO_4_, 2.0 CaCl_2_, 26.0 NaHCO_3_, and 20.0 glucose gassed with 95% O_2_–5% CO_2_, was used for hypotonic stimulation. In some experiments, drugs were dissolved in ACSF in the small chamber to inhibit taurine transporter or anion channel. The supernatant (120 μl) was subsequently collected and stored at -80°C until HPLC analysis. After the samples were derivatized with *o*-phthalaldehyde, the concentrations of amino acid neurotransmitters were determined via reverse-phase HPLC with electrochemical detection. The HPLC system (BAC-300 system; EICOM, Kyoto, Japan) consisted of an EP-300 pump, an ECD-300 electrode and a PowerChrom system (ADInstruments, Sydney, Australia). Separation of amino acid derivatives was achieved using an Eicompack MA-5ODS column (150 mm × 3 mm, EICOM) kept at 30°C. The detection potential was set at +700 mV against an Ag/AgCl reference electrode. The flow rate was 0.5 ml/min, and the sensitivity was set at 64 nA/V full-scale. The mobile phase consisted of (in mM) 86.2 NaH_2_PO_4_.2H_2_O, 13.8 Na_2_HPO_4_.12H_2_O, 0.015 EDTA.2Na, and 30% (v/v) methanol at pH 6.0 and was membrane filtered (0.45 μm) and degassed (DG-300; EICOM) at a flow rate of 0.5 ml/min (EP-300). The amino acids and the derivatives were mixed and allowed to react for exactly 2 min before injection. The amino acid derivatives were then detected electrochemically. Sample concentrations were determined based on comparison of peak areas with that of an external standard.

### D-CYSTEINESULFINIC ACID ADMINISTRATION

Taurine is formed via the oxidation of hypotaurine, an intermediate generated by L-type cysteinesulfinic acid (L-CSA) decarboxylation. L-CSA is formed by the oxidation of cysteine and is the main taurine precursor in rodents. D-CSA is used as a metabolism-resistant, potent and specific inhibitor of L-CSA decarboxylase (Weinstein et al., 1988; Dominy et al., 2004). After electroporation, D-CSA (15 mmol/kg) was administered every 12 h via maternal intraperitoneal injection. As a control group, saline was administered to other mice at the same interval. D-CSA was custom synthesized from D-cysteine using D-cysteine thiosulfonate according to a previously reported procedure (Emiliozzi and Pichat, 1959).

### DRUGS

The following drugs were used: SR95531, bicuculline methiodide, and strychnine (Sigma-Aldrich, St. Louis, MO, USA); CNQX, D-AP5, picrotoxin, bumetanide, and 2-(guanidino)ethanesulfonic acid (GES), CGP55845 (Tocris Bioscience, Bristol, UK); PLGA and TTX (Wako); 4-(2-butyl-6,7-dichlor-2-cyclopentylindan-1-on-5-yl) oxobutyric acid (DCPIB; Santa Cruz Biotechnology, Santa Cruz, CA, USA); 4,4′-diisothiocyanatostilbene-2,2′-disulfonic acid (DIDS; Invitrogen, Carlsbad, CA, USA); and D-cystine (Acros Organics, Fair Lawn, NJ, USA).

### STATISTICS

Unless otherwise indicated, all numerical data are presented as the mean ± SEM. Differences between two groups were assessed using Student’s *t*-test for absolute values and the Mann–Whitney *U* (MWU)-test for normalized values. Comparisons among several groups were performed via one-way ANOVA. The distribution patterns of radially migrating cells were compared using chi-square tests (χ^2^-tests). Differences were considered statistically significant at *P* < 0.05.

## RESULTS

### THE DISTRIBUTION OF RADIALLY MIGRATING CORTICAL PLATE CELLS DOES NOT DIFFER BETWEEN WILD-TYPE AND GAD67-GFP KNOCK-IN MICE

**FIGURE 1:**
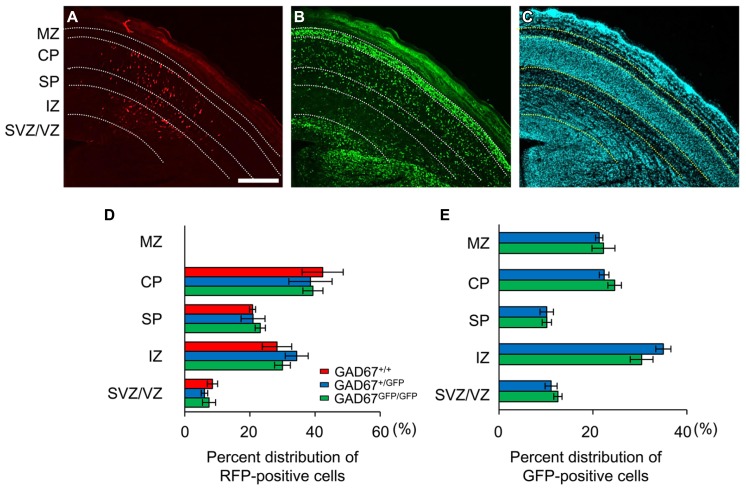
**Distribution patterns of radially migrating neurons and tangentially migrating GABAergic neurons in the cerebral cortex at E17.5**. **(A)** Distribution of radially migrating neurons expressing mRFP (*red*) in a coronal section of the neocortex of a GAD67^+/GFP^ fetus. **(B)** Distribution of GABAergic neurons expressing GFP (*green*) in the same section. **(C)** The section was counterstained with DAPI. The dotted lines in **(A–C)** indicate the boundaries of the proliferative and migratory zones. The scale bar represents 200 μm. MZ, marginal zone; CP, cortical plate; SP, subplate; IZ, intermediate zone; SVZ, subventricular zone; VZ, ventricular zone. **(D)** Percent distributions of RFP-positive cells in the CP, SP, IZ, and SVZ/VZ averaged over five GAD67^+/+^ fetuses, four GAD67^+/GFP^ fetuses, and five GAD67^GFP/GFP^ fetuses. **(E)** Percent distributions of GFP-positive cells in the MZ, CP/SP, IZ, and SVZ/VZ (GAD67^+/+^: *n* = 4; GAD67^+/GFP^: *n* = 5). Error bars indicate the mean ± SEM.

We previously demonstrated that the ambient levels of GABA in the cerebral cortices of heterozygous (GAD67^+^^/GFP^) and homozygous (GAD67^GFP/GFP^) GAD67-GFP knock-in fetuses were 67.8 and 12.7%, respectively, of the level in wild-type (GAD67^+^^/^^+^) fetuses (Morishima et al., 2010). Thus, GAD67-GFP knock-in mice are suitable for investigating the influence of decreased ambient GABA levels on the radial migration of neurons during neocortical development. We performed *in utero* electroporation to label radially migrating neurons with RFP at E14.5. Three days after electroporation (E17.5), we analyzed the distribution of RFP- and GFP-positive cells across the proliferative and the migratory zones of the neocortex in cortical slices. The boundaries of individual zones were determined based on differences in cell density revealed by DAPI counterstaining (**Figure 1C**). RFP-positive cells were mainly distributed in the CP, SP, and IZ in all three genotypes of fetuses (**Figure 1A**). In GAD67^+^^/GFP^ and GAD67^GFP/GFP^ fetuses, GFP-positive cells were also distributed in the MZ (**Figure 1B**). There were no cells expressing both RFP and GFP, confirming that the radially migrating neurons were different from the GABAergic neurons. The RFP- and GFP-positive cells in each region were counted and are presented as the percentages of total RFP- and GFP-positive cells in the neocortex, respectively (**Figures 1D,E**). There were no significant differences in the distribution patterns of RFP- and GFP-positive cells between the genotypes (*P* = 0.925 for RFP-positive cells, **Figure 1D**; *P* = 0.833 for GFP-positive cells, **Figure 1E**; χ^2^-test). Thus, these data suggest that the radial migration of neurons in the neocortex was not affected by decrements in ambient GABA levels.

### THE SENSITIVITY OF GABA_A_Rs IN RADIALLY MIGRATING NEURONS DOES NOT DIFFER BETWEEN WILD-TYPE and GAD67-GFP KNOCK-IN MICE

**FIGURE 2:**
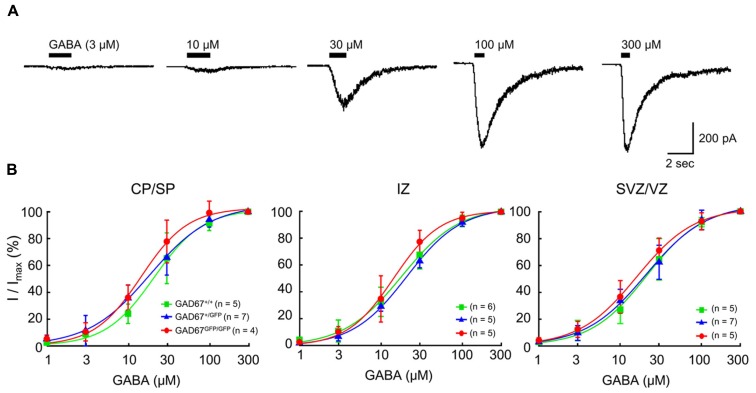
**The sensitivity of GABA_A_Rs to GABA in RFP-positive cells in the E17.5 cerebral cortex does not differ between wild-type and GAD67-GFP knock-in mice**. **(A)** Representative GABA-evoked currents recorded in an RFP-positive cell from a GAD67^+^^/^^+^ fetus. The holding potential (*V*_H_) was -60 mV. The puff application of GABA was stopped immediately after desensitization was initiated. **(B)** Dose–response curves of GABA currents. The EC_50_s of the current responses in GAD67^+^^/^^+^, GAD67^+^^/GFP^, and GAD67^GFP/GFP^ fetuses were 16.23 ± 1.75, 17.46 ± 2.11, and 19.22 ± 2.43 μM, respectively, in the CP/SP (*P* = 0.657 by ANOVA); 16.98 ± 1.80, 20.28 ± 1.13, and 18.53 ± 2.64 μM, respectively, in the IZ (*P* = 0.512); and 16.24 ± 2.61, 18.79 ± 1.66, and 20.39 ± 2.12 μM, respectively, in the SVZ/VZ (*P* = 0.422). The Hill coefficients were 1.36 ± 0.13, 1.26 ± 0.08, and 1.34 ± 0.07, respectively, in the CP/SP (*P* = 0.696); 1.49 ± 0.11, 1.28 ± 0.10, and 1.28 ± 0.08, respectively, in the IZ (*P* = 0.295); and 1.33 ± 0.11, 1.28 ± 0.10, and 1.23 ± 0.16, respectively, in the SVZ/VZ (*P* = 0.891). Error bars: mean ± SEM.

Previous studies have shown that functional GABA_A_Rs are present in developing neocortical cells (LoTurco et al., 1995; Owens et al., 1996). To examine the sensitivity of GABA_A_Rs to GABA in wild-type and GAD67-GFP knock-in fetuses, we examined the currents elicited via puff application of GABA in RFP-positive, radially migrating neurons in all three genotypes. Under whole-cell voltage-clamp conditions at -60 mV, the application of GABA (3–300 μM) produced inward currents in all of the tested RFP-positive neurons (**Figure 2A**). The dose dependence of the peak amplitude of the current on GABA was fitted with a Hill equation (see Materials and Methods), and the dependence was examined in each plate/zone of the neocortex. The EC_50_s and Hill coefficients of the current responses were approximately 18 μM and 1.3, respectively, in all GAD67^+^^/^^+^, GAD67^+^^/GFP^, and GAD67^GFP/GFP^ fetuses, and there were no significant differences in these values between the genotypes or between the plates and zones (**Figure 2B**). Thus, these data indicate that the sensitivity of GABA_A_Rs between the genotypes does not differ at any of the migration stages of radially migrating neurons.

### BLOCKADE OF GABA_**A**_Rs *IN VIVO* INCREASES THE RADIAL MIGRATION SPEED IN GAD67^**GFP/GFP**^ MICE

**FIGURE 3:**
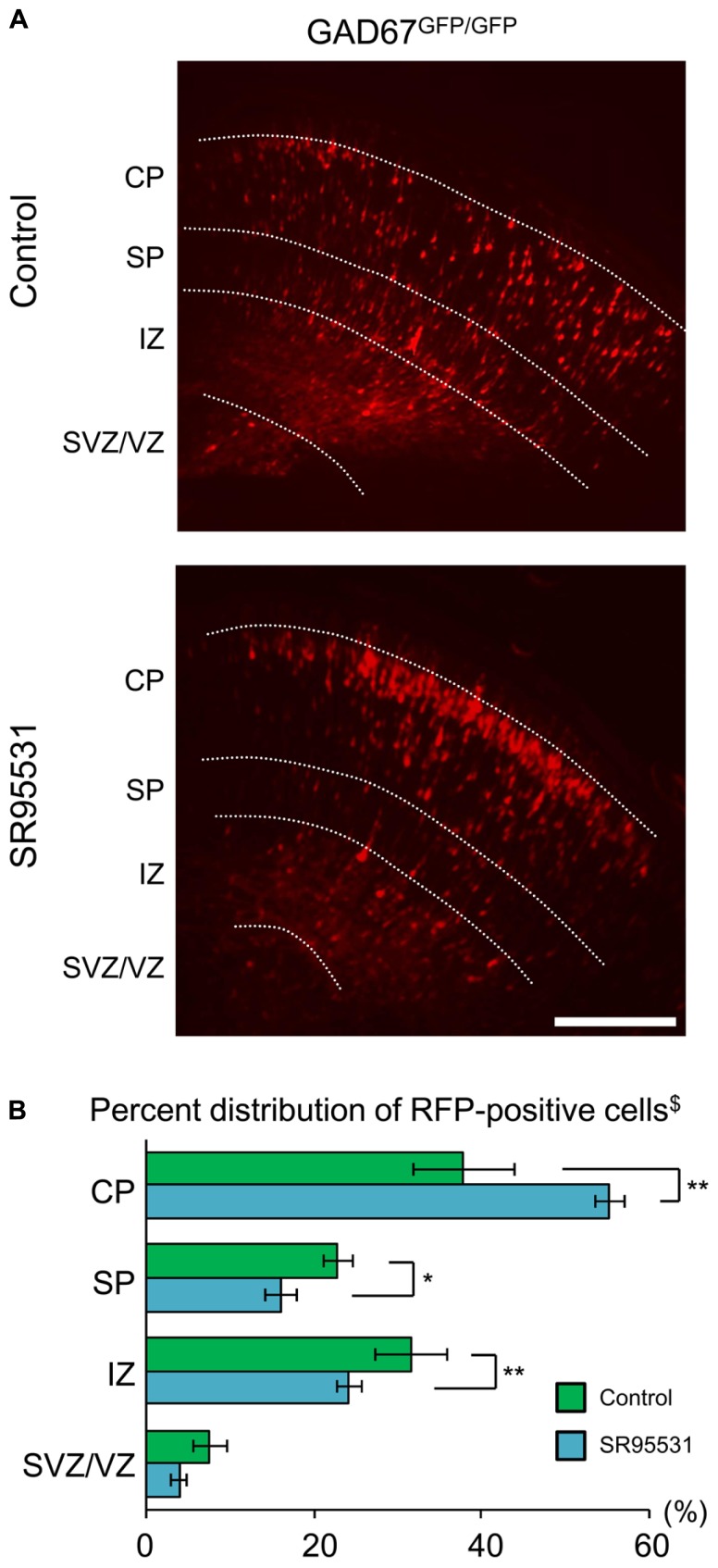
**Sustained administration of a GABA_A_R antagonist facilitates radial migration in GAD67^GFP/GFP^ fetuses**. **(A)** Photomicrographs showing the distribution of RFP-positive cells in Control (upper) and SR95531-treated (lower) GAD67^GFP/GFP^ fetuses at E17.5. PLGA with or without SR95531 was injected into the lateral ventricles of GAD67^GFP/GFP^ fetuses at E14.5, immediately after the electroporation of RFP vectors. **(B)** Proportions of radially migrating cells in the CP, SP, IZ, and SVZ/VZ. The distribution pattern of RFP-positive cells in SR95531-treated GAD67^GFP/GFP^ fetuses was significantly different from that in Control, untreated fetuses (χ^2^-test, ^$^*P* < 0.05; Control: *n* = 6, SR95531-treated: *n* = 6). The percentage of RFP-positive cells was increased in the CP and decreased in the SP and IZ by SR95531 administration (*P < 0.05, **P < 0.01). Error bars: mean ± SEM. Scale bar: 200 μm.

We showed that the distribution pattern and GABA_A_R sensitivity of radially migrating neurons were normal, even in GABA-deficient GAD67^GFP/GFP^ mice (**Figures 1 and 2**). These results suggest the possibility that GABA_A_Rs might not be involved in radial migration. To test this hypothesis, we continuously administered the selective GABA_A_R antagonist SR95531 to GAD67^GFP/GFP^ fetuses via intra-ventricular injection. SR95531-adsorbed PLGA was injected into the lateral ventricles of GAD67^GFP/GFP^ fetuses immediately after *in utero* electroporation at E14.5. Three days after PLGA injection, the distribution pattern of RFP-positive cells was analyzed. SR95531 administration significantly altered the distribution pattern of RFP-positive cells compared with the Control injected with drug-free PLGA (*P* = 0.049 by χ^2^-test, *n* = 6 in Control, *n* = 6 in SR95531; **Figure 3**). The numbers of RFP-positive cells were significantly increased in the CP and decreased in the SP and IZ following SR95531 administration (*P* = 0.001 in CP, *P* = 0.014 in SP, *P* = 0.002 in IZ by MWU-test, *n* = 6 in Control, *n* = 6 in SR95531; **Figure 3B**). This indicates an acceleration of radial migration induced by SR95531 in GAD67^GFP/GFP^ fetuses, suggesting that under normal conditions, GABA_A_Rs are activated and play a role in slowing the radial migration speed, even in GAD67^GFP/GFP^ fetuses, where the ambient GABA level in the neocortex is very low. These data suggest that another endogenous GABA_A_R agonist might activate the GABA_A_Rs of radially migrating cells.

### LOCALIZATIONS OF TAURINE AND TAURINE TRANSPORTERS IN THE MZ AND SP OF THE DEVELOPING CEREBRAL CORTEX AT E17.5

**FIGURE 4:**
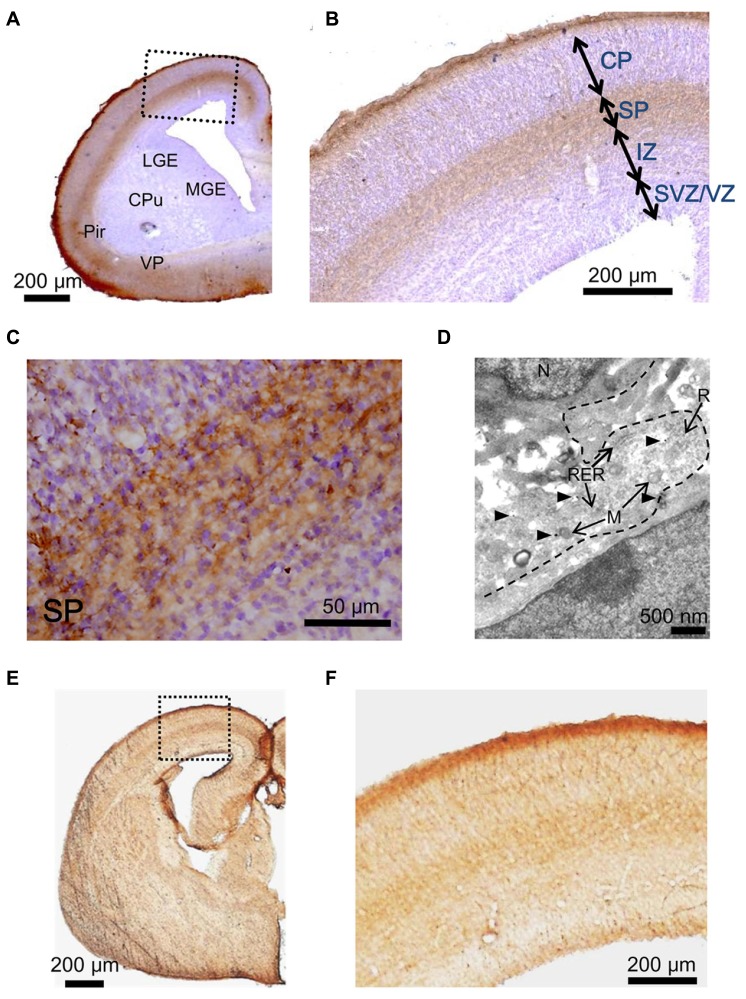
**Immunostaining to detect taurine and the taurine transporter in the cerebral cortices of GAD67^+/+^ fetuses at E17.5**. **(A)** Photomicrograph showing the distribution of taurine in a coronal section of the cortex. The section was counterstained with hematoxylin and eosin (blue). CPu, caudate putamen; LGE, lateral ganglionic eminence; MGE, medial ganglionic eminence; VP, ventral pallidum; Pir, piriform cortex. **(B)** Enlarged view of the boxed area shown in **(A)**. **(C)** Higher magnification view of the SP area shown in **(B)**. **(D)** Electron micrograph of a taurine-immunolabeled section. Arrowheads indicate gold particles. M, mitochondria; N, nucleus; R, ribosome; RER, rough endoplasmic reticulum. **(E)** Photomicrograph of the distribution of the taurine transporter in a coronal section of the cortex. **(F)** Enlarged view of the boxed area shown in **(E)**.

Next, we examined the hypothesis that the GABA_A_R agonist taurine might play a role in regulating radial migration in GAD67^GFP/GFP^ fetuses. To investigate taurine localization in the developing cerebral cortex at E17.5, we performed immunohistochemical analyses in GAD67^+^^/^^+^ mice. Immunoreactivity for taurine was prominent in the MZ and SP (**Figures 4A–C**). To further identify the precise location of taurine in the cells, we next performed immunoelectron microscopic observations using a taurine antibody. Taurine immunoreactivity was present intracellularly and was diffusely located in the cytosol (**Figure 4D**). In the examined specimens, neither presynaptic structures such as synaptic vesicles nor postsynaptic densities were observed.

Taurine is transported into cells by taurine transporters, which have been reported to be expressed in the developing brain (Smith et al., 1992). The localization of taurine transporters in the cerebral cortex was also examined in GAD67^+/+^ fetuses at E17.5. Immunoreactivity for taurine transporters was observed in the MZ and SP, which are the same regions where taurine immunoreactivity was found (**Figures 4E,F**). These data suggest that taurine accumulates in cells in the MZ and SP, where it may be released to activate GABA_A_Rs on radially migrating cells.

### TAURINE EVOKES GABA_**A**_R-MEDIATED CURRENTS IN RADIALLY MIGRATING CELLS

**FIGURE 5:**
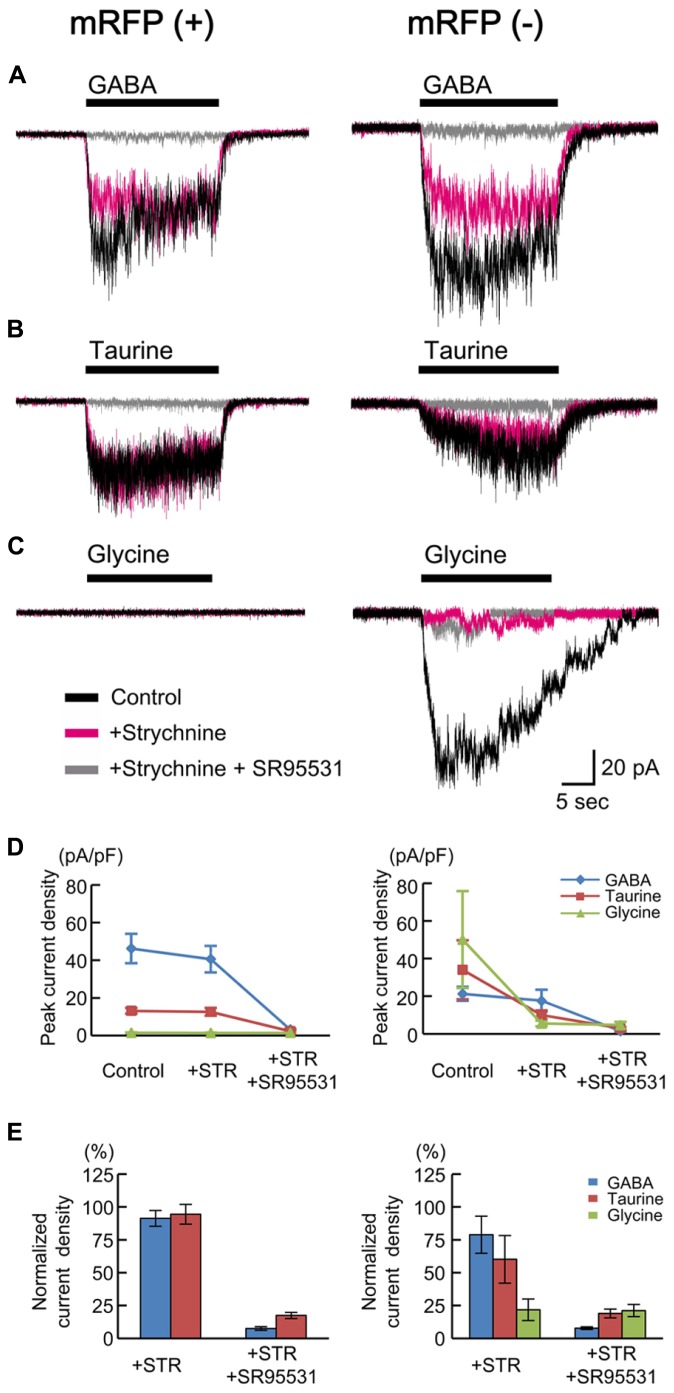
**GABA-, taurine-, and glycine-evoked currents in RFP-positive and -negative cells in GAD67^+/+^ fetuses**. Typical traces of the currents evoked by the applications of 10 μM GABA **(A)**, 10 mM taurine **(B)**, and 3 mM glycine **(C)** in RFP-positive (left panels) and RFP-negative (right panels) cells are shown. Black traces indicate the currents in the absence of blockers; red traces are the currents obtained after the application of 10 μM strychnine through bath perfusion in the same cells; and gray traces are the currents recorded after the further addition of 10 μM SR95531 to the bath solution. *V*_H_ was -60 mV. **(D)** The means ± SEMs of the peak current densities of the agonist-evoked currents in RFP-positive (left panel) and RFP-negative (right panel) cells in the absence and presence of strychnine (STR) and after the addition of SR95531 are plotted. **(E)** Effects of blockers on agonist-evoked currents. The peak current densities in the absence of blockers were normalized to 100%.

Taurine is known to act as an agonist of glycine receptors (GlyRs). GlyRs have been reported to be activated non-synaptically during early neocortical development in rat fetuses (Flint et al., 1998). In addition, taurine may act as a full agonist or a partial agonist of GABA_A_Rs (Dominguez-Perrot et al., 1996). To test whether taurine activates GlyRs or GABA_A_Rs in radially migrating neurons, we compared the currents evoked by the application of GABA, taurine, and glycine in RFP-positive cells located in the SP of the cortices of GAD67^+^^/^^+^ fetuses at E17.5 and examined the effects of blockers of GABA_A_Rs and GlyRs on these currents. The peak amplitude of GABA (10 μM)-evoked inward currents was 46.20 ± 7.78 pA/pF (*n* = 10; **Figures 5A,D**, left), and these currents were not blocked by the selective GlyR antagonist strychnine (10 μM: 91.29 ± 6.01%, *P* = 0.161 by paired *t*-test, *n* = 10; **Figures 5A,D,E**, left), whereas they were blocked by SR95531 (10 μM: 7.54 ± 1.41%, *P* < 0.001, *n* = 9; **Figures 5A,D,E**, left). Indeed, taurine (10 mM) evoked inward currents (13.13 ± 1.57 pA/pF, *n* = 10; **Figures 5B,D**, left), which were also not blocked by strychnine (94.44 ± 7.50%, *P* = 0.455, *n* = 10; **Figures 5B,D,E**, left) but were blocked by SR95531 (17.42 ± 2.32%, *P* < 0.001, *n* = 9; **Figures 5B,D,E**, left). The application of glycine (3 mM) did not produce any currents in RFP-positive cells (1.45 ± 0.18 pA/pF, *n* = 10; **Figures 5C,D**, left). Thus, radially migrating neurons in fetal mouse cortices do not exhibit functional GlyRs, and taurine activates GABA_A_Rs in these neurons.

We also examined the currents in RFP-negative cells for comparison and found that glycine could evoke strychnine-sensitive inward currents in six out of seven recorded cells (50.08 ± 25.71 pA/pF, 21.78 ± 8.20% in the presence of strychnine, *n* = 6; **Figures 5C–E**, right). Thus, some RFP-negative cells express functional GlyRs. GABA and taurine were able to elicit SR95531-sensitive inward currents in the RFP-negative neurons responsive to glycine, but the taurine-evoked currents were also blocked weakly by strychnine in these neurons (60.14 ± 18.06%, *P* = 0.205, *n* = 6; **Figures 5B,D,E**, right), and the GABA-evoked currents were blocked more weakly (78.84 ± 14.10%, *P* = 0.372, *n* = 6; **Figures 5A,D,E**, right). This indicates that the RFP-negative cells possessing GlyRs also respond to taurine.

**FIGURE 6:**
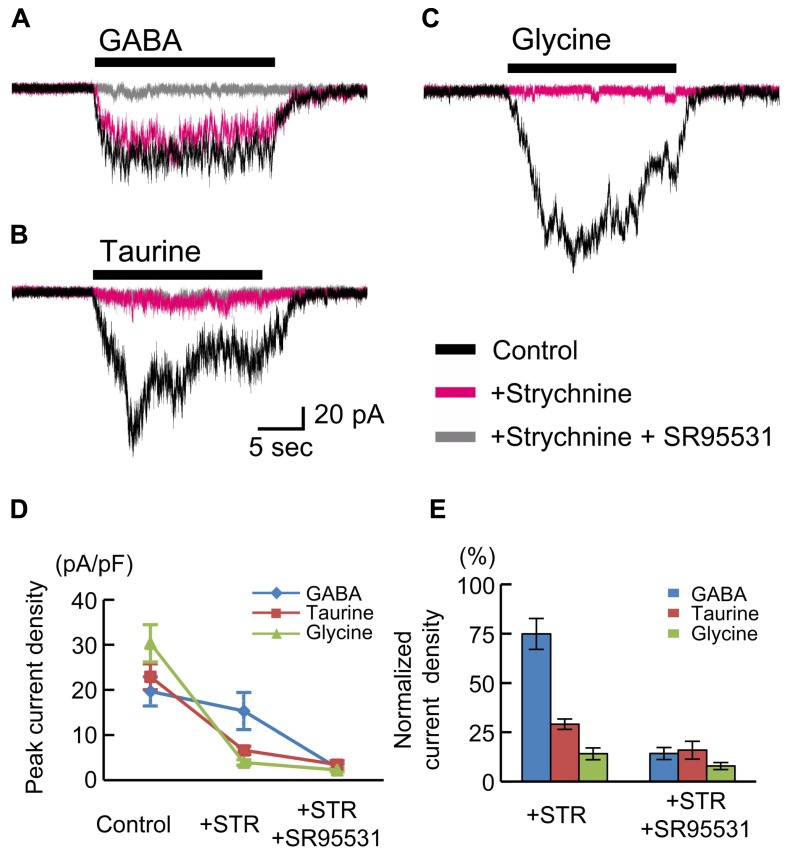
**GABA-, taurine-, and glycine-evoked currents in RFP-positive cells in rat fetuses at E18.5**. Typical traces of the currents evoked by the application of 10 μM GABA **(A)**, 10 mM taurine **(B)**, and 3 mM glycine **(C)** in RFP-positive cells are shown. Black traces indicate the currents in the absence of blockers; red traces are the currents obtained after the application of 10 μM strychnine through bath perfusion in the same cells; and gray traces are the currents recorded after the further addition of 10 μM SR95531 to the bath solution. *V*_H_ was -60 mV. **(D)** The means ± SEMs of the peak current densities in the absence and presence of strychnine (STR) and after the addition of SR95531 are plotted. **(E)** Effects of blockers on agonist-evoked currents. The peak current densities in the absence of blockers were normalized to 100%.

The insensitivity of RFP-positive cells to glycine (**Figures 5C,D**) appears to be inconsistent with findings in rat fetuses (Flint et al., 1998; Nimmervoll et al., 2011). Therefore, we also examined the currents in RFP-positive cells in cortical slices obtained from rat fetuses at E18.5. The peak amplitudes of GABA (10 μM)-evoked inward currents recorded in RFP-positive cells located in the CP of rat cortices were 19.66 ± 3.22 pA/pF (*n* = 6; **Figures 6A,D**), and these currents were not blocked by strychnine (10 μM: 74.92 ± 7.84%, *P* = 0.062 by paired *t*-test, *n* = 6; **Figures 6A,D,E**) but were blocked by SR95531 (10 μM: 14.25 ± 3.07%, *P* < 0.001, *n* = 6; **Figures 6A,D,E**). Taurine (10 mM) also evoked inward currents (22.94 ± 2.87 pA/pF, *n* = 6; **Figures 6B,D**), but these currents were found to be significantly blocked by strychnine (29.09 ± 2.63%, *P* < 0.001, *n* = 6; **Figures 6B,D,E**). Additionally, the application of glycine (3 mM) indeed produced strychnine-sensitive inward currents in RFP-positive cells in rat cortices (30.32 ± 4.14 pA/pF, *n* = 6; blocked by strychnine to 14.13 ± 2.99%, *P* < 0.001, *n* = 6; **Figures 6C,D,E**). Thus, in the rat fetal neocortex, radially migrating neurons express GlyRs (Nimmervoll et al., 2011), and the taurine-evoked inward currents are mainly mediated by these GlyRs.

### ENDOGENOUS TAURINE ACTIVATES GABA_**A**_Rs, PRODUCING TONIC CURRENTS IN RADIALLY MIGRATING NEURONS

**FIGURE 7:**
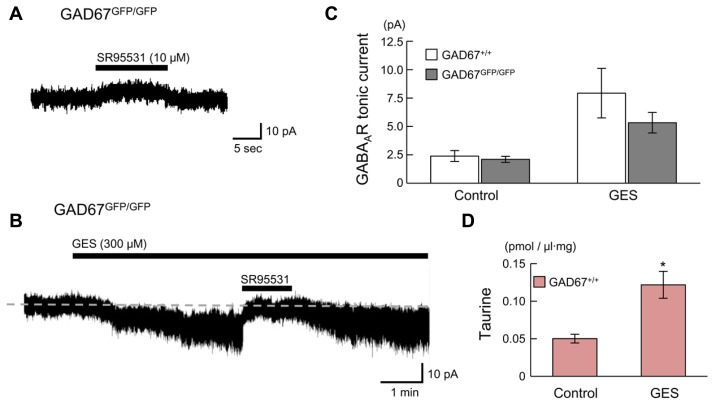
**GABA_A_Rs in radially migrating neurons are activated by endogenous taurine**. **(A)** Typical trace of a GABA_A_R-mediated tonic current in an RFP-positive cell in the SP under normal conditions. *V*_H_ was -60 mV. **(B)** SR95531-sensitive inward current induced by the application of GES. The baseline currents immediately before (for 5 s) the initiation and the cessation of SR95531 application were averaged, to determine the tonic currents based on their difference. **(C)** Averaged tonic current amplitudes before and after GES application. There were no significant differences between the GAD67^+^^/^^+^ (*n* = 5) and GAD67^GFP/GFP^ (*n* = 5) mice. **(D)** HPLC measurements of the taurine concentrations in the cortical slice incubation medium either without (Control, *n* = 5) or with GES (300 μM, *n* = 4). Error bar: mean ± SEM. *P < 0.01.

We next examined whether endogenous taurine activates GABA_A_Rs in radially migrating cells in the fetal mouse neocortex. GABA_A_R-mediated tonic currents were recorded under whole-cell voltage-clamp conditions at -60 mV in RFP-positive cells located in the SP region of the developing cortex at E17.5, and the currents were compared between GAD67^+^^/^^+^ and GAD67^GFP/GFP^ fetuses. The GABA_A_R-mediated components were revealed by the application of 10 μM SR95531 (**Figure 7A**). There was no difference in the GABA_A_R-mediated component observed under normal conditions between GAD67^+^^/^^+^ (*n* = 5) and GAD67^GFP/GFP^ (*n* = 5) fetuses (*P* = 0.609 by Student’s *t*-test; **Figure 7C**, left bars). The membrane capacitance of the recorded cells was similar between the two types of fetuses (GAD67^+^^/^^+^: 11.29 ± 0.97 pF, GAD^GFP/GFP^: 8.57 ± 2.15 pF, *P* = 0.283 by Student’s *t*-test). Application of the taurine transporter inhibitor GES (300 μM) induced an inward shift of the holding currents, which was reversed by the application of SR95531 (**Figure 7B**). The baseline noise of the currents [root mean square (rms) noise level; GAD67^+/^^+^: 1.42 ± 0.09 pA; GAD^GFP/GFP^: 1.80 ± 0.09 pA] was also enhanced by GES treatment (GAD67^+^^/^^+^: 4.58 ± 0.99 pA, *P* = 0.030 by paired *t*-test; GAD^GFP/GFP^: 4.07 ± 0.22 pA, *P* < 0.001 by paired *t*-test) and suppressed by SR95531 (GAD67^+^^/^^+^: 1.83 ± 0.30 pA, *P* = 0.025 by paired *t*-test; GAD^GFP/GFP^: 2.08 ± 0.15 pA, *P* = 0.001 by paired *t*-test). Thus, GABA_A_R activity was increased in the presence of GES, causing an increase in holding currents, while the currents recorded in GAD67^+^^/^^+^ and GAD67^GFP/GFP^ mice showed no significant difference in magnitude (*P* = 0.303, *n* = 5; **Figure 7C**, right bars).

GES has been reported to activate GABA_A_Rs in cerebellar granule cells (Mellor et al., 2000). To confirm whether GES acts on GABA_A_Rs or taurine transporters in radially migrating neurons, we measured ambient taurine levels in cortical slices incubated in medium with or without GES via HPLC. The taurine levels in the cortices of GAD67^+^^/^^+^ fetuses were significantly enhanced in the presence of GES (*P* = 0.004 by Student’s *t*-test; **Figure 7D**). Thus, GES blocked taurine transporters in the cortices, thereby blocking the uptake of extracellular taurine into the cells and increasing ambient taurine levels. These data suggest that endogenous taurine can activate GABA_A_Rs to produce tonic currents in the developing cerebral cortices of both GAD67^+^^/^^+^ and in GAD67^GFP/GFP^ fetuses. Furthermore, we confirmed that the differences in the expression level of GAD67 between the genotypes did not affect the ambient taurine levels (GAD67^+^^/^^+^: 50.21 ± 5.79 fmol/μl·mg, *n* = 5; GAD67^+^^/GFP^: 46.87 ± 3.85 fmol/μl·mg, *n* = 6; GAD67^GFP/GFP^: 45.74 ± 5.16 fmol/μl·mg, *n* = 4; *P* = 0.811 by ANOVA). Thus, the presence of GABA_A_R-mediated tonic currents in GAD67^GFP/GFP^ fetuses under normal conditions with amplitudes similar to those found in GAD67^+^^/^^+^ fetuses (**Figure 7C**, left bars) suggests that taurine, rather than GABA, contributes to the generation of the tonic currents in GAD67^GFP/GFP^ fetuses.

### MATERNAL ADMINISTRATION OF D-CSA DECREASES AMBIENT TAURINE LEVELS AND GABA_**A**_R-MEDIATED TONIC CURRENTS IN BOTH WILD-TYPE AND GAD67 KNOCK-IN FETUSES

If taurine is the major endogenous agonist of GABA_A_Rs in GAD67^GFP/GFP^ fetuses, inhibition of taurine production in the mother would have an effect on radial migration in the fetuses, similar to the effect of blockade of GABA_A_Rs; i.e., facilitation of migration (**Figure 3**). To investigate this hypothesis, we intraperitoneally injected a competitive inhibitor of taurine production, D-CSA, into pregnant mice every 12 h beginning at E14.5, after *in utero* electroporation for cell labeling and examined its effects on ambient taurine levels, GABA_A_R activation and the radial migration of neurons in the fetal neocortices at E17.5.

**FIGURE 8:**
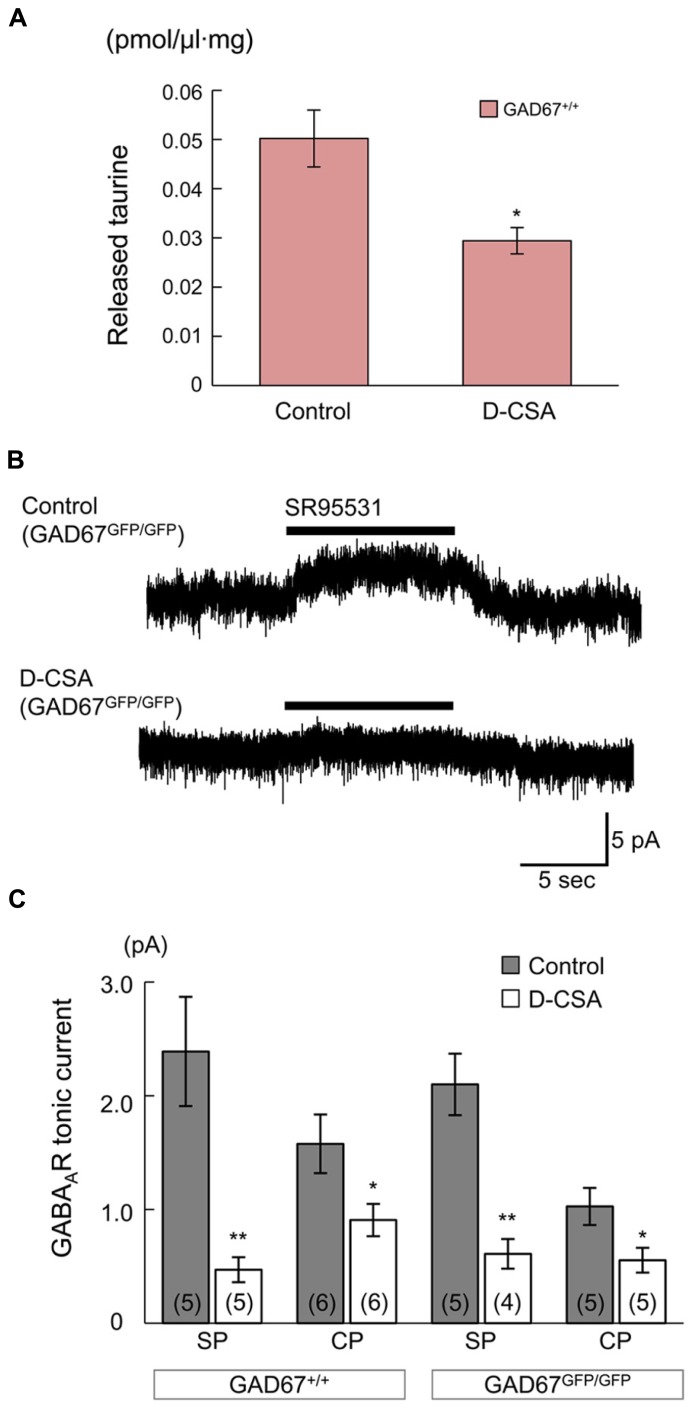
**Effects of maternal D-CSA administration on ambient taurine levels and GABA_A_R-mediated tonic currents in the fetal neocortex**. **(A)** Ambient taurine levels in fetal cortical slices determined via HPLC. E17.5 GAD67^+^^/^^+^ fetuses were obtained from pregnant mice injected with either saline (Control, *n* = 5) or D-CSA (*n* = 5). **(B)** Typical traces of GABA_A_R-mediated tonic currents recorded in RFP-positive neurons in the SP regions of GAD67^GFP/GFP^ fetal cortical slices. The fetuces were obtained from saline- (upper trace) and D-CSA-treated (lower trace) pregnant GAD67^+/GFP^ mice. **(C)** Averaged tonic current amplitudes in the SP and the CP regions of cortical slices obtained from GAD67^+^^/^^+^ (left bar group) and GAD67^GFP/GFP^ (right bar group) fetuses either without (gray bars) or with (white bars) maternal D-CSA injection. The numbers in parentheses indicate the number of samples. *P < 0.05, **P < 0.01. Error bar: mean ± SEM.

First, we determined the ambient taurine levels in cortical slices from GAD67^+/+^ fetuses via HPLC. The taurine levels were significantly decreased by maternal administration of D-CSA (Control: 50.21 ± 5.79 fmol/μl·mg, *n* = 5; D-CSA: 29.42 ± 2.69 fmol/μl·mg, *n* = 5; *P* = 0.012 by Student’s *t*-test; **Figure 8A**). Next, we examined the effects of decreased taurine levels on GABA_A_R-mediated tonic currents in RFP-positive, radially migrating neurons in the SP and CP regions of the cortical slices. In both the SP and CP regions, maternal D-CSA administration significantly reduced the amplitudes of the tonic currents, not only in GAD^GFP/GFP^ fetuses (Control SP: 2.10 ± 0.27 pA, *n* = 5; D-CSA SP: 0.61 ± 0.13 pA, *n* = 4; *P* = 0.002 by Student’s *t*-test; **Figures 8B,C**, third bar group from the left; Control CP: 1.03 ± 0.16 pA, *n* = 5; D-CSA CP: 0.55 ± 0.11 pA, *n* = 5; *P* = 0.042 by Student’s *t*-test; **Figure 8C**, rightmost bar group) but also in GAD67^+^^/^^+^ fetuses (Control SP: 2.39 ± 0.48 pA, *n* = 5; D-CSA SP: 0.47 ± 0.11 pA, *n* = 5; *P* = 0.004 by Student’s *t*-test; **Figure 8C**, leftmost bar group; Control CP: 1.58 ± 0.26 pA, *n* = 6; D-CSA CP: 0.91 ± 0.14 pA, *n* = 6; *P* = 0.046 by Student’s *t*-test; **Figure 8C**, second bar group from the left). There was no difference in the membrane capacitance of the recorded cells observed between Control and maternal D-CSA-treated fetuses (GAD67^+^^/^^+^; 11.29 ± 0.97 pF in Control SP, 12.59 ± 3.19 pF in D-CSA SP, *P* = 0.708 by Student’s *t*-test, 8.58 ± 0.91 pF in Control CP, 7.02 ± 1.22 pF in D-CSA CP, *P* = 0.334 by Student’s *t*-test; GAD^GFP/GFP^; 8.57 ± 2.15 pF in Control SP, 8.50 ± 1.22 pF in D-CSA SP, *P* = 0.979, by Student’s *t*-test, 8.63 ± 1.53 pF in Control CP, 8.86 ± 1.29 pF in D-CSA CP, *P* = 0.913, by Student’s *t*-test). The significant suppression of tonic currents observed in even GABA-rich GAD67^+^^/^^+^ fetuses suggests that taurine plays a major role as an endogenous agonist of GABA_A_Rs, producing tonic currents irrespective of the genotype involved, at least in the SP and CP regions of the fetal cerebral cortex at E17.5.

### RADIAL MIGRATION IS ACCELERATED BY REDUCED AMBIENT TAURINE LEVELS IN GAD67-GFP KNOCK-IN MICE

**FIGURE 9:**
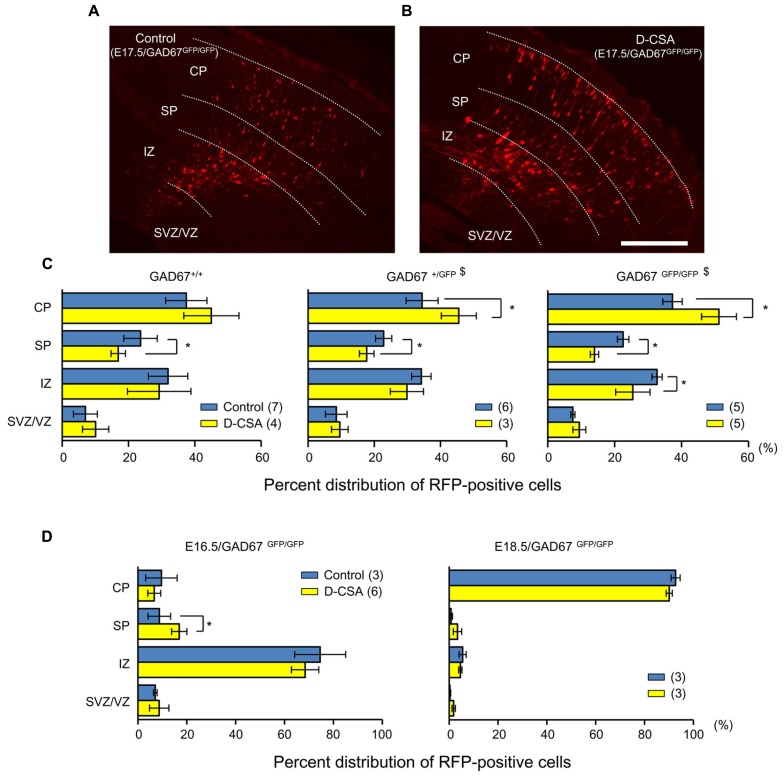
**Effects of maternal D-CSA administration on the distribution of radially migrating neurons in the fetal neocortex**. **(A,B)** Fluorescent micrographs showing the distribution of RFP-labeled radially migrating cells in cortical sections from GAD67^GFP/GFP^ fetuses at E17.5. Saline (Control) **(A)** or D-CSA **(B)** was administered to the mothers every 12 h for 3 days (E14–17). **(C)** The averaged proportions of radially migrating cells in the CP, SP, IZ, and SVZ/VZ regions of cortical slices obtained from GAD67^+^^/^^+^ (leftmost), GAD67^+^^/GFP^ (middle), and GAD67^GFP/GFP^ (rightmost) fetuses at E17.5 with (yellow bars) and without (blue bars) maternal D-CSA administration. **(D)** The averaged proportions of radially migrating cells in cortical slices obtained from GAD67^GFP/GFP^ fetuses at E16.5 (left) and E18.5 (right) either with (yellow bars) or without (blue bars) maternal D-CSA administration. The numbers in parentheses indicate the number of fetuses examined. ^$^*P* < 0.05 by χ^2^-test. *P < 0.05 by MWU-test. Error bar: mean ± SEM. Scale bar: 200 μm.

Next, we examined the effects of maternal D-CSA administration on the radial migration of neurons in the fetal neocortex. The distribution patterns of RFP-positive cells with and without D-CSA treatment were compared in GAD67^+^^/^^+^, GAD67^+^^/GFP^, and GAD67^GFP/GFP^ fetuses. In the GAD67^+^^/GFP^ and GAD67^GFP/GFP^ fetuses, the distribution pattern of RFP-labeled cells was altered significantly by D-CSA administration (*P* = 0.046 in GAD67^+^^/GFP^, **Figure 9C**, middle; *P* = 0.015 in GAD67^GFP/GFP^; **Figures 9A–C**, right; χ^2^-test). In GAD67^+^^/GFP^ and GAD67^GFP/GFP^ fetal cortices, the number of RFP-positive cells was increased in the CP (*P* = 0.023 in GAD67^+^^/GFP^; *P* = 0.012 in GAD67^GFP/GFP^; MWU-test) and decreased in the SP (*P* = 0.040 in GAD67^+^^/GFP^; GAD67^GFP/GFP^; *P* = 0.012) by D-CSA administration. In GAD67^GFP/GFP^ fetuses, the number of RFP-positive cells in the IZ was also significantly reduced (*P* = 0.022). Thus, radial migration in GAD67^+^^/GFP^ and GAD67^GFP/GFP^ fetuses was accelerated by the depletion of taurine. Although the change in the pattern was not significant in GAD67^+^^/^^+^ fetuses (*P* = 0.169; **Figure 9C**, left), a similar trend was apparent. In addition, the distribution patterns of RFP-positive cells with and without D-CSA treatment were compared in GAD67^GFP/GFP^ fetuses at E16.5 and E18.5 and were not found to be significantly altered by D-CSA administration (E16.5: *P* = 0.488, **Figure 9D**, left, χ^2^-test; E18.5, *P* = 0.854, **Figure 9D**, right, χ^2^-test). Thus, the speed of radial migration during the period between E16.5 and E18.5 was increased by D-CSA, as early exit from the SP was apparent at E17.5, although the final distribution of E14.5-born neurons at E18.5 was not disturbed by D-CSA (**Figure 9D**, right). These data suggest that in GAD67-GFP knock-in fetuses, taurine, rather than GABA, regulates the radial migration of neurons by activating GABA_A_Rs and slowing the speed of migration.

### TAURINE ACTIVATES A FRACTION OF GABA_**A**_Rs IN RADIALLY MIGRATING NEURONS

**FIGURE 10:**
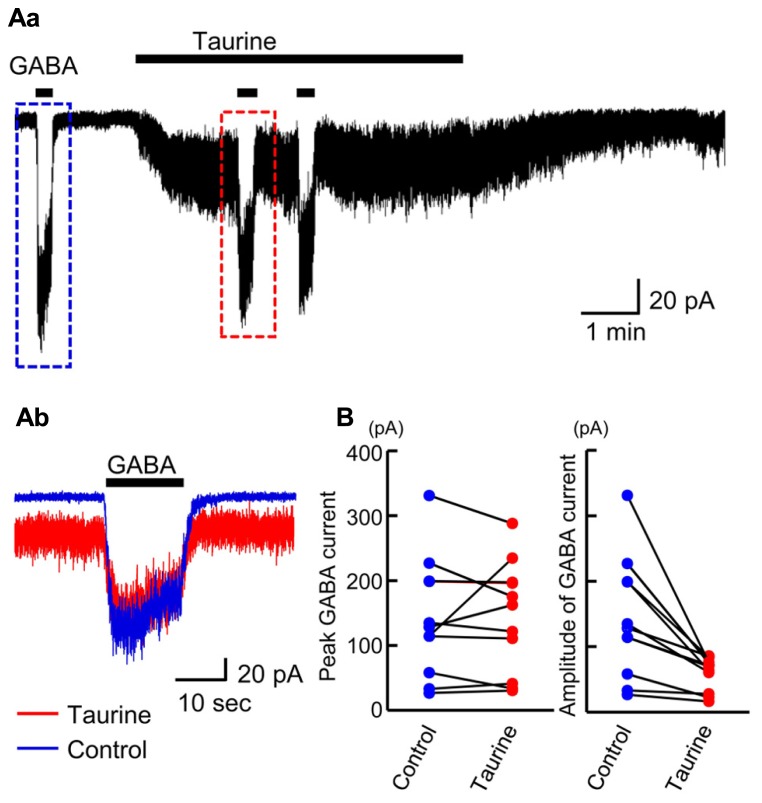
**Comparison of GABA-evoked currents before and during taurine application in radially migrating neurons in the SP region of the fetal neocortex**. **(Aa)** Typical trace of the currents evoked via the puff application of 10 μM GABA before and during bath perfusion of 10 mM taurine in a single RFP-positive neuron. The GABA-evoked currents boxed with dashed colored lines were superimposed in **(Ab)**. *V*_H_ was -60 mV. **(B)** The peak levels of GABA-evoked currents before (Control) and during taurine application (Taurine) are plotted and compared (left, *n* = 10). The levels were not significantly altered by taurine application (*P* = 0.867 by paired *t*-test). The amplitudes of GABA-evoked currents before (Control) and during taurine application (Taurine) are plotted and compared (right). The amplitudes were significantly altered by taurine application (*P* = 0.004 by paired *t*-test).

The affinity of GABA_A_Rs for taurine was recently reported to depend on the subunit composition of GABA_A_Rs (Kletke et al., 2013). To examine how large a fraction of GABA_A_Rs are activated by taurine in radially migrating neurons, we compared the currents evoked by GABA and taurine in the same radially migrating neuron in the SP region of GAD67^+^^/^^+^ fetal cortical slices. For this purpose, we first recorded the currents evoked by the topical application of 10 μM GABA, followed by bath application of taurine at 10 mM. Taurine perfusion induced inward currents that were much weaker than those evoked by GABA, while taurine did not induce significant current desensitization during its perfusion (**Figure 10Aa**). When GABA was again applied in the presence of taurine, the amplitudes of the GABA-evoked inward currents were 49.97 ± 5.83% (*n* = 10) of those prior to taurine perfusion (**Figures 10Ab,B**, right). The peak levels of the GABA-evoked currents observed during GABA application were unchanged by taurine perfusion (**Figures 10Ab,B**, left; *P* = 0.867 by paired *t*-test, *n* = 10). Thus, taurine activates only a fraction (~49.97%) of GABA_A_Rs, with a much lower affinity than GABA in radially migrating neurons.

### TAURINE IS RELEASED THROUGH VOLUME-SENSITIVE ANION CHANNELS IN THE DEVELOPING CEREBRAL CORTEX

Finally, we examined how taurine is released in the developing cerebral cortex. Some studies have reported that the taurine release induced by hypotonic shock in adult brain tissues can be blocked by anion channel blockers (Schousboe et al., 1991; Oja and Saransaari, 1992; Haskew-Layton et al., 2008). Thus, volume-sensitive anion channels are likely to be responsible for this taurine release (Haskew-Layton et al., 2008). To determine whether such anion channels are involved in taurine release in the fetal cerebral cortex, we examined the effects of anion channel blockers and a hypotonic medium on the release of taurine from acute cortical slices obtained from GAD67^+^^/^^+^ fetuses at E17.5.

**FIGURE 11:**
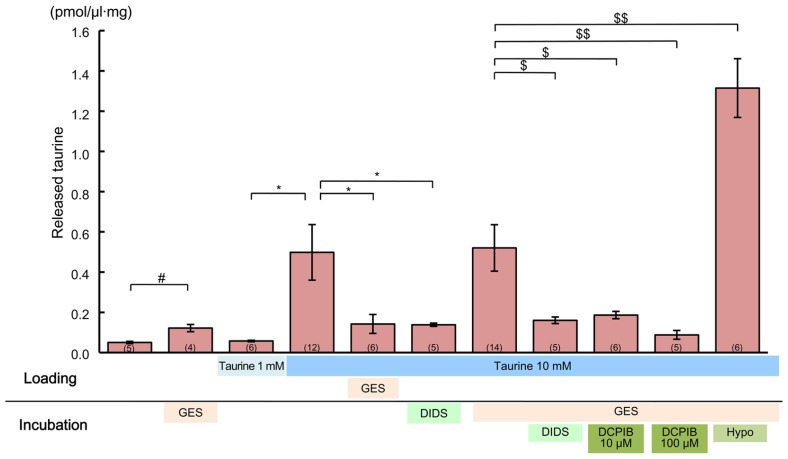
**Effects of anion channel blockers on taurine release in the developing neocortex**. Each bar represents the taurine concentration determined via HPLC in the measurement medium containing cortical slices from GAD67^+^^/^^+^ fetuses at E17.5, either without or with the inhibitors indicated below the bar (“incubation”). The loading conditions, either without or with taurine (1 or 10 mM) and/or GES (300 μM), before the measurements were performed are also indicated just beneath the bar (“loading”). DIDS (1 mM) and DCPIB (10 or 100 μM) were dissolved in incubation media. The numbers in parentheses at the bottom of the bars indicate the number of samples. One sample was obtained from one fetus. ^#^*^$^*P* < 0.05, ^$$^*P* < 0.01. Error bar: mean ± SEM.

To observe these effects clearly, first we preloaded the slices with 10 mM taurine by preincubating them in taurine-containing medium for 1 h. This was expected to enhance the intracellular taurine content, thereby increasing the taurine release flux from the cells. Indeed, the ambient taurine level measured via HPLC after preloading was greatly enhanced (**Figure 11**, fourth bar from the left). This enhancement was suppressed by adding the taurine transporter blocker GES (300 μM) to the preincubation medium (**Figure 11**, fifth bar from the left). Preloading with 1 mM taurine had no detectable effect (**Figure 11**, third bar from the left), suggesting that the rate of taurine uptake by taurine transporters under these loading conditions was balanced by the rate of taurine release from the cells. In addition to preloading the cortical slices with 10 mM taurine, we added GES during the period of taurine level measurement to isolate the taurine release flux. However, GES did not further enhance the measured taurine level (**Figure 11**, fifth bar from the right).

Application of a global inhibitor of anion channels, DIDS (1 mM), to the measurement medium markedly reduced taurine release (**Figure 11**, fourth bar from the right). This reduction was still clearly observable, even in the absence of GES treatment (**Figure 11**, sixth bar from the right). Treatment with a more specific inhibitor of volume-sensitive anion channels, DCPIB (10 and 100 μM), also markedly suppressed taurine release in a dose-dependent manner (**Figure 11**, second and third bars from the right). Moreover, the taurine release was markedly enhanced in the hypotonic medium, which is the most effective activator of volume-sensitive anion channels (**Figure 11**, rightmost bar). Thus, taurine must be released through volume-sensitive anion channels in the developing cerebral cortex.

## DISCUSSION

In the present study, we showed that radial migration in the developing mouse cerebral cortex was modulated by GABA_A_R activation in ambient GABA-deficient GAD67-GFP knock-in mice. Ambient taurine, which abundantly expressed in the SP and MZ, acts as an endogenous agonist of GABA_A_R to tonically activate GABA_A_Rs and attenuate the speed of radial migration.

GABA_A_Rs have been reported to regulate neuronal migration in several cell types and are known to act as a stop signal for radially migrating neurons in the developing cortex (Owens and Kriegstein, 2002; Heck et al., 2007; Manent and Represa, 2007; Wang et al., 2013; for a review, see Egawa and Fukuda, 2013). However, the effect of GABA_A_R activation on neuronal migration has mostly been investigated *in vitro* using GABA_A_R antagonists (Behar et al., 1996, 1998, 2000; Bolteus and Bordey, 2004). In this study, radially migrating cells, labeled via *in utero* electroporation at E14.5, were identified in the cerebral cortex at E17.5. We found that the distribution pattern of the radially migrating cells was normal, even in GABA-deficient GAD67^GFP/GFP^ fetuses. In addition, electrophysiological recordings demonstrated that the GABA_A_R sensitivities of radially migrating cells did not differ among the different genotypes of GAD67-GFP knock-in fetuses. These results are consistent with a previous report showing that GAD65/67 double knock-out embryonic mouse brains do not show any histological abnormality (Ji et al., 1999). Furthermore, we demonstrated that sustained blockade of GABA_A_Rs via SR95531 administration accelerated radial migration in the cerebral cortex of GABA-deficient GAD67^GFP/GFP^ fetuses. These findings imply that activation of GABA_A_R indeed affects radial migration, but that GABA_A_Rs are not necessarily activated by endogenous GABA.

### FUNCTIONAL EXPRESSION OF GABA_**A**_R, BUT NOT GlyR, IN RADIALLY MIGRATING CELLS IN THE FETAL MOUSE NEOCORTEX

GABA-induced inward currents were observed in all recordings obtained from RFP-labeled cells, which is consistent with previous studies demonstrating the presence of functional GABA_A_Rs in these cells (LoTurco et al., 1995; Owens et al., 1996). RFP-positive, radially migrating cells showed GABA_A_R-mediated currents in response to taurine application. Interestingly, none of the RFP-positive cells in which recordings were made showed glycine-induced currents. These results suggest that the GABA_A_Rs, but not GlyRs, on radially migrating cells were activated by taurine in the fetal mouse neocortex. Functional expression of GlyRs has been reported in the fetal and neonatal rat cortex (Malosio et al., 1991; Flint et al., 1998; Nimmervoll et al., 2011). Accordingly, in E18.5 rat fetuses, we found that radially migrating cells labeled via *in utero* electroporation 3 days before recordings were performed exhibited glycine-induced inward currents (**Figure 6**). In addition, in mouse fetuses, some of RFP-negative cells that were presumably generated earlier than the RFP-positive cells responded to the application of glycine by producing large currents. Considering these results, the expression of functional GlyRs might differ between rats and mice. In mice, GlyRs may be expressed after the migratory period of corticogenesis, in contrast to those in rats (Nimmervoll et al., 2011).

The functional properties of GABA_A_Rs and the affinity of taurine are subunit dependent. Previous studies have found that taurine acts on a specific subtype of GABA_A_R containing the β_2_ subunit (Bureau and Olsen, 1991; Kash et al., 2004). Kletke et al. (2013) also reported that the efficacy of taurine is independent of the α subunit type and is instead determined by the β subunit type based on recombinant studies. Furthermore, δ-containing receptors are more potently and efficiently gated by taurine than γ-containing receptors (Jia et al., 2008; Kletke et al., 2013). Considering these findings, taurine is likely to potently activate α_x_β_1/2_δ subunit-containing GABA_A_Rs. GABA_A_R subunits α_2_–α_5_, β_1_–β_3_, γ_1_–γ_2_, and δ are expressed in immature cortical cells, and these subunits are developmentally regulated (Gambarana et al., 1991; Araki et al., 1992; Laurie et al., 1992; Cheng et al., 2006; Peden et al., 2008; Cuzon Carlson and Yeh, 2010). Although the expression of GABA_A_R subunits in radially migrating cells remains unclear, it is possible that they may express the components that mediate the actions of taurine.

### AMBIENT TAURINE TONICALLY ACTIVATES GABA_**A**_R AND MODULATES RADIAL MIGRATION IN THE FETAL CEREBRAL CORTEX

Previous studies have shown that tonic activation of GABA_A_R is evident in certain embryonic neurons before synapse formation has taken place (Valeyev et al., 1993; LoTurco et al., 1995; Owens et al., 1999; Farrant and Nusser, 2005; Cancedda et al., 2007). While we also demonstrated that GABA_A_Rs were tonically activated in the fetal cerebral cortex, GABA_A_R-mediated tonic currents were detected even in GABA-deficient GAD67^GFP/GFP^ fetuses. We found that taurine and its transporter are expressed at particularly high levels in the SP and MZ of the fetal cerebral cortex and that depletion of taurine through maternal D-CSA administration greatly reduces the recorded tonic currents, not only in the SP but also in the CP, in both GAD67^+^^/^^+^ and GAD67^GFP/GFP^ fetuses. Moreover, although it did not reach statistical significance, there was a trend of smaller tonic currents observed in the CP compared to the SP, even under control (D-CSA-untreated) conditions (**Figure 8C**). This might reflect the differences in ambient taurine levels between the SP and CP sensed by GABA_A_Rs. These findings suggest that in the cerebral cortex of fetal mice, taurine might play a major role as an endogenous agonist of GABA_A_Rs and that the GABA_A_Rs in radially migrating cells are activated tonically by ambient taurine, rather than GABA. However, our results do not fully exclude the possibility that some component of GABA_A_Rs is activated by endogenous GABA. The affinity of GABA_A_Rs for GABA is more than three orders of magnitude higher than for taurine (Kletke et al., 2013), which was also indicated by our results (**Figure 10**). Therefore, even though the applied D-CSA treatment decreased the basal taurine level in the fetal cerebral cortex by only 40% (**Figure 8A**), it is possible that the component of tonic GABA_A_R currents that remains after D-CSA treatment (**Figure 8C**) might be due to the action of endogenous GABA to some extent.

The amplitude of tonic GABA_A_R currents in the SP, which were found to be mostly caused by taurine action, was approximately 2.5 pA in both GAD67^+^^/^^+^ and GAD67^GFP/GFP^ fetuses (**Figures 7C and 8C**). However, these currents were recorded in the presence of a high intracellular Cl^-^ concentration (122 mM), introduced through whole-cell patch pipettes. As the intracellular Cl^-^ level under physiological conditions must be lower than that in our experiments, the tonic currents that occur under physiological conditions must be smaller than those we measured because of the weaker driving force for Cl^-^ movement. In previous reports, the resting membrane potential and the reversal potential for GABA_A_R-mediated currents measured by gramicidin-perforated patch-clamp recordings were reported to be -65 and -43 mV, respectively, in rat CP cells at E19 or E18.5 (Owens et al., 1996; Inoue et al., 2012). Assuming conditions similar to these previous reports, the amplitude of tonic currents we measured at the holding potential of -60 mV with an Cl^-^ equilibrium potential of -2 mV would correspond to ~0.9 pA. The membrane resistance of the SP cells estimated in the present study, based on the holding current level at -60 mV (10.08 ± 0.79 pA, *n* = 5), was approximately 6 GΩ. Thus, the 0.9 pA tonic currents may depolarize the cells by 5.4 mV. Nevertheless, because Cs^+^ salts were used in our pipette solution to block K^+^ channels, the 6 GΩ membrane resistance must be an overestimated value, and the degree of depolarization elicited by tonic currents in SP cells under physiological conditions would therefore be no greater than 5 mV.

When the taurine-mediated tonic GABA_A_R currents were reduced via maternal D-CSA administration, the distribution pattern of RFP-positive cells was significantly changed, especially in GAD67^GFP/GFP^ fetuses, indicating an increase in the fraction of RFP-positive cells in the CP and a reciprocal decrease in the SP and IZ at E17.5 (**Figures 9A–C**). This pattern mimicked the change in distribution caused by GABA_A_R blockade (**Figure 3**). These results clearly indicate that the reduction of GABA_A_R activity in the SP caused by taurine depletion accelerates the exit of radially migrating neurons from the SP. Thus, taurine slows the speed of radially migrating neurons across the SP through activating GABA_A_Rs in these neurons. In the cortices of GAD67^+^^/GFP^ fetuses, in which the ambient GABA level is reduced by 32% (Morishima et al., 2010), a similar change in distribution was elicited by taurine depletion, while the change observed in GAD67^+^^/^^+^ fetuses was subtle (**Figure 9C**). As such, the ambient GABA levels could also regulate radial migration, presumably through activating the GABA_A_Rs in regions other than the SP.

The mechanism underlying the regulation of the speed of migrating neurons via taurine-mediated GABA_A_R activation has not yet been resolved. It has been demonstrated in immature cortical neurons that GABA_A_R activation is required to maintain intracellular oscillatory Ca^2^^+^ signaling (Behar et al., 1996; Owens et al., 1996; Garaschuk et al., 2000; Heck et al., 2007). Thus, the stop signal for migration generated by taurine is also expected to be mediated by the Ca^2^^+^ signaling. By contrast, in the developing cerebellum, the migration of granule neurons has been reported to be stopped by the loss of intracellular Ca^2^^+^ signaling (Kumada and Komuro, 2004). Hence, the role of intracellular Ca^2^^+^ signaling in neuronal migration seems to differ between the cortex and the cerebellum. Nevertheless, the notion that the action of taurine in the cerebellum also maintains Ca^2^^+^ signaling in granule cells through activating GABA_A_Rs would be consistent with a previous report indicating a delay in granule cell migration in taurine-deficient kittens (Sturman et al., 1985).

Even when the radial migration of neurons in the developing cortex was accelerated by taurine depletion at E17.5, the destination of the migrating neurons at E18.5 was not affected by taurine depletion (**Figure 9D**). This suggests that the effect of taurine-mediated GABA_A_R activation on radial migration is temporary, and a different process might be involved in the final maturation of the cortex. Nevertheless, it is possible that the early exit of migrating neurons from the SP caused by taurine depletion may affect final circuit formation and/or the function of the neurons after birth in some manner. To examine this possibility, it will be necessary to follow the effect of taurine depletion on the maturation of the cortex in GAD67^+/GFP^ mice in detail even after birth because the GAD67^GFP/GFP^ genotype is lethal immediately after birth due to severe cleft palate, which has been well established in GAD67 knock-out mice (Asada et al., 1997).

### AMBIENT TAURINE IS TAKEN UP BY TAURINE TRANSPORTERS AND RELEASED VIA VOLUME-SENSITIVE ANION CHANNELS

Taurine transporters are highly expressed in the neocortex (Smith et al., 1992). These transporters belong to a family of Na^+^ and Cl^-^-dependent neurotransmitter transporters that mediate the uptake of taurine into cells under resting conditions (Molchanova et al., 2004; Tappaz, 2004). Because taurine uptake is driven by the membrane gradient of Na^+^ and Cl^-^, taurine transporter activity can be reversed by stimuli that alter this gradient (Oja et al., 1985; Oja and Kontro, 1987; Takuma et al., 1996). In the present study, the ambient taurine level in fetal cortical slices was increased via the application of GES. In addition, taurine release from 10 mM taurine-loaded cortical slices was decreased by GES application during the taurine loading period. Furthermore, GABA_A_R-mediated tonic currents of SP cells were enhanced by GES application. These results are compatible with the normal taurine transporter function of extracellular taurine uptake. Thus, ambient taurine is taken up by taurine transporters in the mouse fetal neocortex.

In the present study, we observed ambient taurine levels in the fetal cerebral cortex via HPLC. The released taurine concentration was 45.7–50.2 fmol/μl·mg, while no released GABA was detectable. As our HPLC system is able to detect GABA at the 0.05 fmol/μl·mg, level (Morishima et al., 2010), the taurine levels in our study were more than 1,000 times higher than those of GABA in the cerebral cortex of E17.5 mice. Furthermore, in taurine loading experiments, the released taurine concentration was increased by loading with 10 mM taurine but was not changed by 1 mM taurine loading. These data suggest that the ambient taurine concentration in the fetal cerebral cortex may be in the millimolar range.

We showed that taurine was expressed in the SP and MZ of the cerebral cortex at E17.5 through immunohistochemical analysis. Immunoelectron microscopic analysis demonstrated that taurine was located within immature neurons, whereas it was not detectable in presynaptic structures. These results imply that the release of taurine may be controlled in a non-vesicular manner, which is in accord with previous studies suggesting that taurine can be released as an osmolyte through non-vesicular systems in neurons and glial cells (Calvert and Shennan, 1998; Flint et al., 1998; Mongin et al., 1999; Mulligan and MacVicar, 2006). Previous studies have also shown that taurine can be released via volume-sensitive anion channels (Fugelli and Thoroed, 1986; Jackson and Strange, 1993; Hall, 1995; Shennan, 1999; Haskew-Layton et al., 2008). However, there are limited data on the release of taurine in the fetal nervous system, despite its important role in functional development (Sturman et al., 1985; Palackal et al., 1986); non-synaptic and hypo-osmotic taurine release has been observed in the immature cortex of rats (Flint et al., 1998; Kilb et al., 2008). Our data showed that administration of DIDS, a broad Cl^-^ channel blocker, and DCPIB, a specific volume-sensitive anion channel blocker, blocked taurine release, while a hypotonic medium stimulated taurine release. These results provide evidence that taurine release in the fetal cerebral cortex is mediated by volume-sensitive anion channels (Jackson and Strange, 1993; Shennan, 1999; Molchanova et al., 2004; Haskew-Layton et al., 2008).

The involvement of volume-sensitive anion channels in the radial migration of neurons is an interesting issue. Volume-sensitive anion channels are typically activated in response to cell swelling (Okada, 1997), but they may also be activated without swelling when certain types of chemical mediator or transmitter act on the cell (Liu et al., 2009; Okada et al., 2009). Moreover, it has recently been demonstrated in mouse astrocytes that the transmitter-induced activation of volume-sensitive anion channels is locally controlled via regions of high concentrations of intracellular Ca^2^^+^ in the immediate vicinity of Ca^2^^+^-permeable ion channel pores, or so-called “Ca^2^^+^ nanodomains” (Akita and Okada, 2011; Akita et al., 2011). Therefore, if the volume-sensitive anion channels located on the leading edges of migrating neurons are activated in response to taurine-induced intracellular Ca^2^^+^ signaling in a similar manner to that observed in astrocytes, a net efflux of Cl^-^ and K^+^ should ensue according to their electrochemical gradients, which should accompany an obligatory water efflux. This may facilitate shrinkage of the leading edge and slow the migrating neurons (Schwab et al., 2012). Furthermore, taurine release through the activated channels may send synchronizing signals to adjacent neurons. Exploring the mechanism of the activation of the volume-sensitive anion channels and their roles in the fetal neocortex in detail would therefore shed light on the mystery of fetal brain development, which needs to be addressed in future studies.

## CONCLUSION

We provide new evidence that ambient taurine in the mouse fetal cerebral cortex modulates radial migration. Ambient taurine exhibits a relatively wide distribution with relatively high levels in the SP and is released via volume-sensitive anion channels to tonically activate GABA_A_Rs and slow radial migration in the developing cerebral cortex.

## Conflict of Interest Statement

The authors declare that the research was conducted in the absence of any commercial or financial relationships that could be construed as a potential conflict of interest.
